# The Subjective Well-Being of School Children. The First Findings from the Children’s Worlds Study in Poland

**DOI:** 10.1007/s12187-015-9312-8

**Published:** 2015-03-26

**Authors:** Dorota Strózik, Tomasz Strózik, Krzysztof Szwarc

**Affiliations:** 1 Department of Statistics and Demography, The Poznan University of Economics, Poznań, Poland; 2 Department of Business Activity, The Poznan University of Economics, Poznań, Poland

**Keywords:** Children’s Worlds, Subjective well-being, Poland

## Abstract

The paper presents the first findings of the children’s subjective well-being survey in Poland, which was conducted among representative sample of over 3000 pupils aged 8, 10 and 12 years from Wielkopolska region in spring 2014. The study is a part of International Survey of Children’s Well-being (ISCWeB) - Children’s Worlds, developed by the International Society for Child Indicators (ISCI). The main purpose of the ISCWeB project is to gain a broad knowledge of children’s lives, their relationships with family members and friends, daily activities, time use and, in particular, their own perceptions and evaluations of their well-being. A particular attention in this paper is paid to the children’s subjective well-being including overall satisfaction with life, measured with use of different psychometric scales, eg. the single item scale on Overall Life Satisfaction (OLS) or the five-item Students Life Satisfaction Scale (SLSS5). Along with overall well-being of the children, it is very important to study various domains of their well-being. In the paper we took into consideration children’s evaluation of their five important life domains: family, school, friends, living environment and self.

## Introduction

One of the symptoms of a high standard of development of each country is the care taken to ensure an adequate level of well-being (WB) and quality of life (QOL) to all citizens, and above all to children. In the times of demographic changes it is frequently stressed that the understanding for needs and rights of the youngest generation is crucial as investing in children means investing in the society they will form and contribute to in the future (James and James 2012, p. 59). But the children are not only the “future adults”. Understanding the children’s well-being in terms of their future (e.g., their education or potential employability) risks losing sight of their current life situation whilst the childhood is a stage with its own sociological characteristics (Ben-Arieh 2008, p. 6). An adult-centred perspective disregards the value of childhood itself, postponing children’s well-being to a later generation. But child subjective well-being is a measure of well-being in the here-and-now (Bradshaw et al. 2013, p. 620) and it does not mean (only) well-becoming.

In order to ensure an adequate level of children’s well-being, we need to understand what child well-being is and how it should be measured and analysed (Axford 2009, p. 372). Subjective well-being (SWB) refers to a person’s cognitive and affective evaluations of his or her life, including both emotional reactions as well as cognitive judgements of satisfaction (Diener et al. 2002, p. 63). Similarly, child SWB should be understood as an individual conviction of a young person about the degree of accomplishment of his/her living needs, approached in terms of satisfaction, happiness, fears and apprehensions. According to this concept there exists no other more direct and more valid method to evaluate the level of child living standard than turning directly to those involved. Children are key informants of their lives and relevant agents in providing data on the realities they experience (Casas et al. 2013, p. 436).

Although the best source of information for studying children’s well-being would be the children themselves (Ben-Arieh 2005, p. 581), in practice research on children’s WB from their own perspective is limited. Much research focuses on the child QOL from adult (expert or parental) perspective (Ben-Arieh and Shimon 2014, p. 101), confusing child well-being with adult opinions of child well-being. Article 12. of the Convention of the Right of the Child (CRC) guarantees every child the right to form his or her own opinions, to be listened to and the right to their views being taken into account. However, the low reliability and validity of data obtained from children are often used as an excuse to avoid collecting such data (Casas 2011, p. 564), whereas the children, when given the chance, have both the capacity and ability to participate in research about their lives (Fattore et al. 2009, p. 75).

Despite numerous challenges, a recognition of children as a distinct group (Fattore et al. 2007, p. 11) and understanding for children’s rights to social participation and for their new role in measuring and monitoring their own well-being is clearly growing. However, so far most studies on child well-being have been focused on specific, small populations. Very few studies refer to large samples, and cross-national comparisons are scarce (Casas 2011, p. 562).

The article presents the first findings of the children’s SWB survey in Poland, which was conducted among a representative sample of pupils aged 8, 10 and 12 years from Wielkopolska (Greater Poland) region in spring 2014 as a part of International Survey of Children’s Well-being (ISCWeB). A particular attention in this paper was paid to the children’s SWB including overall satisfaction with life, measured with use of different psychometric scales. Along with overall well-being of the children, it is very important to study various domains of their well-being (Cummins and Lau 2005; Land et al. 2007; Rees et al. 2010). In the paper we took into consideration children’s evaluation of their five important life domains: family, school, friends, living environment and self (Seligson et al. 2003).

## The Children’s Worlds Study

The main purpose of the ISCWeB project is to gain a broad knowledge of children’s lives, their relationships with family members and friends, daily activities, time use and, in particular, their own perceptions and evaluations of their well-being. The ISCWeB is a unique initiative that deepens our understanding of the worldview through the eyes of young people – the children’s world. As the project takes note of the children’s rights, particularly of a child’s right to be heard, it was fundamental to the methodological approach of this study to place the children centrally as research participants (ISCWeB 2013).

Over 45,000 children from 15 countries participated in the current phase of the survey. Since many countries all over the world contributed to this study, it will help to fill a considerable gap regarding actual, international and comparative data that are important in assessing children’s well-being.

Our study used a set of anonymous, self-report questionnaires which were checked during piloting with children of different age groups and socioeconomic backgrounds. The ISCWeB questionnaire was translated into Polish. To ensure the questionnaire dovetailed with the ISCWeB, a back translation technique was used. The children filled out either electronic or traditional paper version of the questionnaires.

The research met the proper ethical guidelines and rules, and received the approval of the appropriate institutes. The survey was confidential and anonymous. All the children were provided with a choice as to withdraw from the study for any reason or not to answer any particular questions. Congruent arrangements were made to ensure that children could fill out the questionnaire in privacy.

### Procedure

The Children’s Worlds study was conducted among primary school pupils aged from 8 to 12 from Wielkopolskie voivodship. The scheme of selecting a representative group of respondents was based upon multistage, stratified random sampling. The prepared sampling frame was a list of primary schools which were assigned weights proportional to their size. The minimum value of the sample in the research project was established at the level of 1000 children from each of the differentiated age groups (8, 10, and 12 years). Because schools were selected with probabilities proportional to the total number of class groups, only one class group for each children’s age group (from 2nd, 4th and 6th grade) was randomly selected from each of the 65 sampled schools. Expecting that children’s well-being differs due to the place of residence, a point was made that children from individual regions of the voivodship and those from rural and urban schools, public and non-public, should be properly represented in the sample.

According to the Polish law a parental consent is not obligatory. Nonetheless, some schools decided that it was required, which consequently decreased the number of potential respondents. The absence of parental consent was responsible for the fact that finally the survey included 3272 pupils from 64 schools. After checking the collected material as regards contents and formal requirements, 3157 respondents were finally qualified for further analysis (1021 8-year-olds, 1119 10-year-olds, and 1017 12-year-olds).

### Sample

The study of children’s well-being was carried out among primary school children, from grades 2, 4, and 6. This means that, apart from a few exceptions, the age of respondents invited to participate the study fell within 8–9 years (2nd grade), 10–11 years (4th grade) and 12–13 years (6th grade).

Out of 3157 school children participating in the study 48 % were girls. Taking into account the type and location of schools taking part in the study, it was established that children from urban schools constituted 57.7 % of all respondents, whereas only one out of 30 pupils attended non-public school. Nearly all young respondents (99.5 %) reported they were born in Poland. Over 99 % of all respondents were living in a household with their own family and 85.5 % of them were living in a full family (both with a mother and a father).

## Instruments

There is a number of specific overall life satisfaction measures that have been constructed for assessing child and adolescent well-being as well as general scales that can be used on the whole population (Gilman and Huebner 2000; Casas 2011). In our study we used three psychometric scales: a single-item scale on Overall Life Satisfaction (OLS), a reduced version of the Students’ Life Satisfaction Scale (SLSS-5), and an adapted version of the Brief Multidimensional Students’ Life Satisfaction Scale (BMSLSS) (Table 1).

**Table 1:** Psychometric scales used in the research

Instruments	Response scales	Items included
OLS	5-point smiley faces scale 11-point scale, from *Not at all satisfied* (0) to *Totally satisfied* (10)	How satisfied are you with your life as a whole?
SLSS-5	5-point scale, from *I don’t agree* to *Totally agree* 11-point scale, from *Not at all agree* (0) to *Totally agree* (10)	My life is going well
		My life is just right
		The things in my life are excellent
		I have a good life
		I have what I want in life
BMSLSS	5-point smiley faces scale 11-point scale, from *Not at all satisfied* (0) to *Totally satisfied* (10)	Satisfaction with your family life
		Satisfaction with your friends
		Satisfaction with your school experience
		Satisfaction with your own body
		Satisfaction with the area where you live

### OLS

It is the simplest single-item, but important measure of overall life satisfaction (Campbell et al. 1976). In our study, we asked the children how satisfied they were with their life as a whole. For the purpose of analysis, the responses obtained on a 5-point faces scale (8-year-olds) or an 11-point scale from “*not at all satisfied*” to “*totally satisfied*”(10- and 12-year-olds) were transformed into a value from 0 to 100.

### SLSS

We used in the study a reduced version of Huebner (1991) Student’s Life Satisfaction Scale, which was successfully tested in earlier studies (e.g., Rees et al. 2010; Casas et al. 2012). The scale was based on five statements about children’s overall life satisfaction (Table 1). The children aged 8 responded to the questions using a 5-point scale with a range from “*I do not agree*” to “*totally agree*”, whereas 10- and 12-year-olds used an 11-point scale ranging from “*not at all agree*” to “*totally agree*”. We formed the total SLSS scale by adding the scores of five items and transforming the sum from 0 to 100. The internal consistency of the SLSS-5 ranged in our sample, depending on the age group, from 0.89 to 0.95 (Cronbach’s α).

### BMSLSS

The next measure applied in the study was an adapted version of the Brief Multidimensional Student Life Satisfaction Scale (Huebner 1994; Seligson et al. 2003). The BMSLSS consisted of five items, each representing one of the five life domains: family, school, friends, living environment and self. We calculated a version of this scale using the questions about children’s satisfaction with their family life, friends, school experience, local area and body (Table 1). The satisfaction was measured on a 5-point faces scale (8-year-olds) or an 11-point scale from “*not at all satisfied*” to “*totally satisfied*” (10- and 12-year-olds). The five items were summed to create a total life satisfaction score and transformed so that it was from 0 to 100. Cronbach’s α of the BMSLSS in our sample lied between 0.64 and 0.76.

## Data Analysis

Data analysis entailed, aside from descriptive analysis, *t*-tests and analysis of variance ANOVA to examine the differences between boys and girls, children from rural and urban schools and between three age groups. A correlation analysis and a multiple regression analysis were used to examine associations between overall life satisfaction measures and the importance of BMSLSS domains in determining children’s global life satisfaction.

## Results

### Overall Life Satisfaction

Generally speaking, the degree of children’s satisfaction with their life is high, as it is indicated by the values of instruments used in the study (the OLS, the SLSS and the BMSLSS) which exceed on average 85 points. The highest mean values for all scales were observed among 8-year-olds (over 90 points each time). The worst assessment of their lives was provided by 12-year-olds (Table 2). The conducted analysis of variance ANOVA confirmed significant differences in the assessment of children’s life satisfaction in the studied age groups (*F*_*OLS*_ = 72.86, *p* < .001; *F*_*SLSS*_ = 52.78, *p* < .001; *F*_*BMSLSS*_ = 95.37, *p* < .001). On the other hand, *post hoc* comparisons by means of Games-Howell test showed the absence of significant differences in the average SWB assessments of 8- and 10-year-olds (*p*_*OLS*_ = .07; *p*_*SLSS*_ = .051; *p*_*BMSLSS*_ = .052).

**Table 2:** Children’s SWB measures

Age group	Total		Boys		Girls		Urban		Rural	
	Mean	%^a^	Mean	%	Mean	%	Mean	%	Mean	%
	OLS									
8 year-old	93.46	5.16	93.50	4.60	93.48	5.51	92.73	6.37	94.46	3.50
10 year-old	91.90	5.51	91.88	5.25	92.51	5.13	91.08	6.13	93.04	4.64
12 year-old	84.30	10.74	86.39^*^	9.56	82.18^*^	11.82	83.89	11.25	84.86	10.06
	SLSS									
8 year-old	90.01	5.92	89.70	5.62	90.51	5.96	89.48	6.80	90.74	4.72
10 year-old	88.13	5.96	88.14	5.34	88.67	5.96	87.49	6.12	88.98	5.74
12 year-old	81.44	10.51	82.37	9.90	80.50	11.20	81.13	10.69	81.85	10.27
	BMSLSS									
8 year-old	91.05	0.83	90.40^**^	0.60	91.86^**^	1.12	89.96^*^	1.07	92.54^*^	0.51
10 year-old	89.82	1.69	89.91	1.83	89.83	1.55	88.97^*^	1.87	91.02^*^	1.44
12 year-old	83.26	4.97	83.89	4.57	82.64	5.40	82.99	5.62	83.64	4.08

^a^Percentage of children with low well-being

*t*-tests: ^*^ *p* < .01, ^**^ *p* < .05

Nearly 11 % of the sixth grade pupils scored 50 or less out of 100 on the OLS scale, and we classify these children in the study as having low SWB. The same measure indicates that the low level of overall life satisfaction is typical of every twentieth 8- and 10-year old. A similar share of the children with low well-being was obtained on the basis of the SLSS (Table 2). On the other hand, assessments based on the BMSLSS indicate that the share of pupils who are dissatisfied with their life as a whole is considerably smaller (8-year-olds: 0.83 %, 10-year-olds: 1.69 %, 12-year-olds: 4.97 %).

The average level of life satisfaction is similar for both genders (Table 2). Statistically significant SWB disproportions between boys and girls appeared only among 12-year-olds and concerned the OLS values (higher for boys) and among 8-year-olds, concerning the BMSLSS values (higher result for girls). Moreover, small differences (the biggest for 12-year-olds) occurred in the fraction of both genders of children with low life satisfaction.

The average marks obtained on the scales applied indicate that life satisfaction is slightly higher in children from the rural schools, however statistically significant differences appeared only on the BMSLSS among 8-year-olds (the average of 89.96 in cities as compared with 92.54 in villages) and 10-year-olds (the average of 88.97 in cities compared with 91.02 in villages).

### Satisfaction with Different Life Domains

The analysis of how much children are satisfied with particular aspects of their lives, leads to a conclusion that although they have a relatively positive attitude to particular aspects, some differences are visible in their assessments (Table 3).

**Table 3:** Children’s satisfaction with various life domains

Satisfaction with:	Total		Boys		Girls		Urban		Rural	
	Mean	%^a^	Mean	%	Mean	%	Mean	%	Mean	%
	8-year-olds^b^									
Family life	3.73	5.04	3.68^**^	6.76	3.78^**^	3.16	3.71	5.31	3.75	4.67
Friends	3.62	5.39	3.62	5.89	3.62	4.91	3.56^*^	6.60	3.69^*^	3.73
School exp.	3.45	14.44	3.42	16.70	3.48	11.69	3.38^*^	16.53	3.54^*^	11.59
Own body	3.71	4.87	3.71	5.19	3.72	4.59	3.70	4.40	3.73	5.51
Local area	3.68	5.70	3.65	6.66	3.72	4.49	3.62^*^	6.87	3.77^*^	4.08
	10-year-olds									
Family life	9.38	4.47	9.35	5.02	9.42	3.73	9.37	3.90	9.39	5.25
Friends	8.73	8.25	8.76	8.02	8.74	7.75	8.63^**^	8.66	8.86^**^	7.71
School exp.	8.59	8.73	8.57	9.18	8.66	7.60	8.49	9.35	8.72	7.88
Own body	8.97	7.66	9.03	7.72	8.92	7.69	8.89	7.61	9.08	7.75
Local area	9.06	6.43	9.08	6.37	9.06	6.41	8.97^**^	7.53	9.19^**^	4.90
	12-year-olds									
Family life	9.00	6.30	8.99	6.98	9.00	5.71	8.97	6.66	9.03	5.82
Friends	8.31	11.06	8.31	11.00	8.32	11.04	8.27	12.14	8.38	9.60
School exp.	8.05	15.07	7.83^**^	17.88	8.25^**^	12.27	8.01	15.09	8.09	15.04
Own body	7.81	17.69	8.34^**^	12.42	7.28^**^	22.81	7.76	18.12	7.87	17.11
Local area	8.34	11.82	8.42	10.29	8.26	13.09	8.38	12.36	8.29	11.11

^a^Percentage of children with low satisfaction

^b^Scale 0–4

*t*-tests: ^*^ *p* < .01, ^**^ *p* < .05

Out of the five differentiated domains (the BMSLSS items) the most favourable marks in all age categories were obtained for family life. Higher average assessments were observed among girls (although this difference was statistically significant only for 8-year-olds) and in rural areas. In general, about 5 % of the children expressed dissatisfaction with the discussed aspect (scored midpoint or below), and the biggest share of children with low family life satisfaction was obtained in the group of 12-year-olds (6.30 %).

Regardless of their gender, children aged 8 and 10 are well satisfied with their bodies, but this domain received a much worse assessment from 12-year-olds, particularly from teenage girls (Table 3). It is symptomatic that nearly 23 % of 12-year-old girls expressed dissatisfaction with their physical appearance, whereas among younger children percentage of those dissatisfied with their bodies reached the level of approximately 6 %.

Similarly, children’s satisfaction with friends decreases along with age. One out of nine 12-year-olds expressed dissatisfaction with his/her friends while in the group of 10-year-olds this number reached little more than 8 % and among 8-year olds only one person out of twenty was dissatisfied with this aspect of his/her life. When taking into account school location, statistically significant differences were found between younger (8- and 10-year-olds) children from rural and urban schools. City children assess their friends relatively worse. Differences between boys and girls in their average assessment of friends were slight.

Out of the five aspects under discussion, school experience received the worst assessment, regardless of age. Low satisfaction with school life was indicated by approximately 15 % of 8- and 12-year-olds and by nearly 9 % of 10-year-olds. In each age group average assessments given by boys were lower than those given by girls, although this difference was statistically significant only among the oldest children (the average of 7.83 for boys as compared with 8.25 for girls). While analysing differences in the children’s satisfaction with their school according to its location, higher average marks for school experiences are characteristic of the children from rural schools and the biggest differences in those marks were observed among the 2nd grade pupils.

The local area where the children live was assessed rather positively, although their satisfaction with this aspect decreases along with a child’s age (Table 3). Among the oldest children the percentage of persons expressing low satisfaction with the local area reached the level of nearly 12 % (over 13 % for girls) and thus it was more than two times higher than among 8-year-olds. Moreover, in the group of 8- and 10-year-olds the degree of satisfaction with the local area was higher among pupils from rural schools. Those differences were statistically insignificant among the 6th grade pupils.

### The Structure of Children’s Well-Being

In accordance with our expectations, the correlation between psychometric scales used to study children’s overall life satisfaction is moderate or strong, which confirms the convergent validity of those measures. Among 8-year-olds the BMSLSS correlates 0.526 with the OLS and 0.495 with the SLSS. The OLS and the SLSS correlate 0.470. Among older children the correlation between the differentiated measures is even stronger, reaching the level between 0.688 and 0.774 (Table 4).

**Table 4:** Correlations of BMSLSS life domains with overall SWB measures

	8-year-olds		10-year-olds		12-year-olds	
	OLS	SLSS	OLS	SLSS	OLS	SLSS
SLSS	0.467		0.725		0.718	
BMSLSS	0.526	0.495	0.688	0.728	0.708	0.774
Family life	0.354	0.385	0.470	0.565	0.481	0.634
Friends	0.315	0.277	0.461	0.515	0.489	0.537
School exp.	0.313	0.402	0.484	0.489	0.486	0.541
Own body	0.433	0.320	0.585	0.536	0.585	0.577
Local area	0.305	0.271	0.426	0.467	0.445	0.494

All five life domains included on the BMSLSS show significant correlation with other scales (the OLS and the SLSS). In all of the three age groups, the strongest correlation with the OLS was observed for satisfaction with own body (Table 4). However, when taking into account correlation with the SLSS, school experience turned out to be most significant among 8-year-olds (0.402), and family life among older children (0.565 and 0.634 respectively).

Among 8-year-olds two successive positions as regards the strength of correlation with overall SWB were taken by family life and friends (OLS) or family life and own body (SLSS). As far as 10-year-olds are concerned, school experience was ranked second and family life was ranked third in association with the OLS, whereas the second and the third positions as regards the strength of correlation with the SLSS were occupied by: own body and friends. As concerns the oldest children, the second and the third positions were taken by the following domains: friends and school experience (OLS) or own body and school experience (SLSS). For all age groups the weakest correlation with the OLS and the SLSS concerned satisfaction with the area the children live in (Table 4).

The importance of BMSLSS life domains in determining children’s overall life satisfaction was confirmed by a multiple regression analysis (Table 5). On the basis of standardized coefficients of regression (beta) it was established that all life domains contribute in a significant way to child WB. In each of the age groups the OLS is most strongly affected by satisfaction with one’s own body (0.300 among 8-year-olds, 0.367 among 10-year-olds and 0.349 among 12-year-olds). The SLSS, in turn, is most strongly influenced by school experience in the group of 8-year-olds (0.258), and by family life among 10- and 12-year-olds (0.320 and 0.338 respectively).

**Table 5:** Regression of BMSLSS life domains on the OLS and the SLSS

	OLS		SLSS	
	Beta	*p* value	Beta	*p* value
	8-year-olds			
Satisfaction with family life	0.160	0.000	0.221	0.000
Satisfaction with friends	0.136	0.000	0.052	0.115
Satisfaction with school exp.	0.111	0.000	0.258	0.000
Satisfaction with own body	0.300	0.000	0.148	0.000
Satisfaction with local area	0.136	0.000	0.079	0.014
Adjusted R^2^	0.300	0.255		
	10-year-olds			
Satisfaction with family life	0.238	0.000	0.320	0.000
Satisfaction with friends	0.100	0.000	0.165	0.000
Satisfaction with school exp.	0.164	0.000	0.159	0.000
Satisfaction with own body	0.367	0.000	0.242	0.000
Satisfaction with local area	0.106	0.000	0.153	0.000
Adjusted R^2^	0.501		0.547	
	12-year-olds			
Satisfaction with family life	0.184	0.000	0.338	0.000
Satisfaction with friends	0.178	0.000	0.175	0.000
Satisfaction with school exp.	0.131	0.000	0.141	0.000
Satisfaction with own body	0.349	0.000	0.286	0.000
Satisfaction with local area	0.148	0.000	0.148	0.000
Adjusted R^2^	0.512		0.622	

All the BMSLSS items together explained ca. 30 % of the OLS and 26 % of the SLSS among 8-year-olds. Amid 10-year-olds it was 50 % of the OLS and 55 % of the SLSS, whilst among 12-year-olds - 51 % and 62 %, respectively.

## Discussion

In the article we present the initial findings from the children’s SWB survey conducted in Poland among the children aged 8–12 years. The study shows that the young people at this age declare fairly high satisfaction with their lives. On the other hand, the share of children whose assessment of their life was low reached (depending on age category and the applied measure) the level of 0.8 % (BMSLL for 8-year-olds) to even 10.7 % (OLS for 12-year-olds). Calculating the average, this yields almost 13 thousand children on the regional and 135 thousand on the national scale of youngsters who are not satisfied with their life (with the assumption that in other voivodships of Poland the tendencies are similar to those in Wielkopolska).

When examining different life domains, the study reports that the aspect of life which the children are most satisfied with (regardless of age and sex) is their family life. The study confirms previous results (e.g., Casas et al. 2013; The Children’s Society 2014) about the importance of family relations as one of the crucial contributors to children’s SWB.

For school communities particularly important can be the fact that much worse marks are given by the pupils mainly to their school experience. More research on different school life aspects (e.g., school violence, relations with classmates and with teachers) is needed to better understand the reasons of this situation and to provide guidelines for school climate improvement.

Various national and international studies have found children’s and adolescents’ life satisfaction decreasing with age (e.g., Casas 2011; Currie et al. 2012; Goswami 2014). The results of our research are similar to these findings and show lower SWB as children get older. For all analysed life aspects older children felt significantly less satisfied than younger children. Those differences are mainly visible in the comparison of 12-year-olds with their younger colleagues and concern, above all, satisfaction with their own bodies.

The results suggest that both girls and boys tend to display a high level of overall life satisfaction with a mixed pattern of small (in absolute terms) gender differences. Taking into account various life domains according to sex, the differences are more visible. For example, as in the previous studies (e.g., The Children’s Society 2014), it was found that girls were more satisfied than boys with their school experience and less happy about their body, particularly as they got older. It is worth to emphasize the scale of this phenomenon. According to our research, there is a considerable group of children with low self-esteem and body concerns. These children need special attention as body image dissatisfaction results not only in low SWB but often leads to unhealthy practices, like eating disorders, smoking or alcohol abuse (Currie et al. 2012, p. 93).

Considering territorial differences, the study shows that children from rural area schools had slightly higher levels of overall SWB whichever measure was used. Pupils from rural schools were also more satisfied with their school experience and friends as well as with the local area (mainly the youngest children).

Both different values of the three psychometric scales and the explored structure of children’s well-being confirm that these measures refer to the same construct, but from a different perspective. The study shows that the overall SWB is something more than satisfaction with different life domains (albeit important) because “the whole is more than the sum of its parts” (Casas et al. 2013, p. 444).

Initial findings from the Polish part of ISCWeB presented in the article are intentionally brief and the study has some important limitations. The children’s SWB is a very complex phenomenon and the study focuses on, beside overall satisfaction with life, only on five children’s life domains (family, friends, school, living environment and self). Future studies need to look at different aspects of young people’s lives (e.g., material situation, feeling of safety, health or time use) and their influence on children’s SWB. There is also need to take into consideration some, not included in this article, various socio-demographic characteristics (e.g., material deprivation, family structure, number of siblings) found in previous studies (e.g., Dinisman et al. 2012; Goswami 2014) to be important for explaining children’s well-being. Finally, we didn’t examined in our study the effect of life events and changes in children’s lives (such as moving home, moving school, changes in family structure or in family economic status) on their SWB. For policymakers and educationalists as well as for the general public in Poland, it would be particularly important to recognise the phenomenon of so called “Euro-orphanage” and explore if and how economic emigration of parents influences children’s subjective well-being.

In conclusion, these article can be considered as an initial study of Polish children’s well-being based on their own voices. The study shows that the children are a very reliable source of the information about their lives albeit from a different perspective. The children can teach us a lot about how we can better their childhood. Therefore, utilizing a huge potential of the Children’s Worlds survey, a detailed analysis needs to be and will be done for a better understanding of children’s lives, both in Poland and in other countries.
